# USING CREATIVE NARRATIVES AND VISIONS OF LATER LIFE TO QUEER GERONTOLOGICAL SCHOLARSHIP

**DOI:** 10.1093/geroni/igae098.1946

**Published:** 2024-12-31

**Authors:** Sarah Jen

**Affiliations:** University of Kansas, Lawrence, Kansas, United States

## Abstract

Queering gerontology calls for more diverse and inclusive visions of old age, including those that resist normative ways of being. LGBTQ people have particular contributions to make to this project given their experiences of non-normative life events, sequences, and desires. This study presents a secondary qualitative data analysis, drawing from two existing datasets (13 life reviews with older bisexual-identified women and 40 creative narratives published in Bi Women Quarterly). The analysis examines queer and non-normative visions and experiences of aging from the perspective of queer and trans individuals across the life course. The findings are examined through the lens of three principles of queer gerontology, illustrating the potential of queer and creative visions of aging to reimagine the potential of gerontological scholarship. Notably, the creation and dissemination of queer narratives of aging and later life deepen visibility and awareness of diverse and marginalized experiences while also offering new and creative ways of imagining later life for all populations. The examined narratives engage critical perspectives, often dispelling normative ways of being through camping up, resisting, or queering notions of self, kinship, life and death, and the narrative form. Narratives also illustrate the engagement of multiple epistemologies, creating potential for more creative modes of inquiry, including creative writing and imagery, in envisioning later lives. These findings indicate the potential of revisiting existing data through the lens of queer gerontology principles to illuminate the possible forms queer gerontological scholarship could take, both empirical and conceptual.

